# Social Participation in Relation to Technology Use and Social Deprivation: A Mixed Methods Study Among Older People with and without Dementia

**DOI:** 10.3390/ijerph17114022

**Published:** 2020-06-05

**Authors:** Sophie N. Gaber, Louise Nygård, Anna Brorsson, Anders Kottorp, Georgina Charlesworth, Sarah Wallcook, Camilla Malinowsky

**Affiliations:** 1 Division of Occupational Therapy, Department of Neurobiology, Care Sciences and Society, Karolinska Institutet, 14183 Huddinge, Sweden; sophie.gaber@ki.se (S.N.G.); anna.brorsson@ki.se (A.B.); anders.kottorp@mau.se (A.K.); sarah.wallcook@ki.se (S.W.); camilla.malinowsky@ki.se (C.M.); 2 Faculty of Brain Sciences, University College London, London WC1E 6BT, UK; 3 Faculty of Health and Society, Malmö University, 20506 Malmö, Sweden; 4 Research Department of Clinical, Educational and Health Psychology, University College London, London WC1E 6BT, UK; g.charlesworth@ucl.ac.uk; 5 Research and Development Department, North East London NHS Foundation Trust, Maggie Lilley Suite, Goodmayes Hospital, Essex IG3 8XJ, UK

**Keywords:** digital accessibility, technologies for aging, information and communication technologies, social participation, dementia

## Abstract

Social participation is a modifiable determinant for health and wellbeing among older people; however, social participation is increasingly dependent on technology use. This study investigated social participation in relation to Everyday Technology use and social deprivation of the living environment, among older people with and without dementia in the United Kingdom. Sixty-four people with dementia and sixty-four people without dementia were interviewed using standardized questionnaires: The Participation in ACTivities and Places OUTside Home Questionnaire and Everyday Technology Use Questionnaire. A mixed methods approach integrated statistical analyses and content analysis of free-text responses, through data visualizations. Small, statistically significant associations were found between social participation and Everyday Technology use outside home, for participants with dementia (*R_s_* = 0.247; *p* = 0.049) and without dementia (*R_s_* = 0.343; *p* = 0.006). A small, statistically significant association was identified between social participation and social deprivation in the living environment, among only participants with dementia (*R_s_* = 0.267, *p* = 0.033). The content analysis and graphical joint display revealed motivators, considerations that require extra attention, and strategies for managing social participation. The results underline how Everyday Technology use can be assistive to social participation but also the need to consider social deprivation of the living environment, especially among people with dementia.

## 1. Introduction

The World Health Organization (WHO) [1] classifies social participation as social determinant of health, and according to research, it is a modifiable determinant for health and quality of life among older people. The WHO [1] states that whilst policymakers cannot create participation, more can be done to create “spaces” that enable and encourage social participation, especially involving vulnerable and marginalized communities, such as older people with dementia. Social participation is linked to numerous positive health outcomes, including: increased physical functioning and improved psychosocial wellbeing [2], reduced social isolation [3], reduced risk of functional disability [4], prevention of cognitive decline [5] and other noncommunicable diseases such as cardiovascular diseases or cancers [6], as well as the promotion of active and healthy ageing [7]. Benefits of social participation are relevant for older people who are considered susceptible to the negative effects of social isolation and loneliness on their health and wellbeing [3]. By identifying barriers and motivators to social participation, vulnerable and marginalized communities may be empowered to access the “spaces” and places in society where they can harness the benefits of social participation on their health and quality of life [1].

Living within an increasingly digitalized society means interacting with technology, in order to engage in social participation [8]. Research shows that digital technology can enhance social participation among older people [9], reinforce existing social relationships [10], and mitigate social isolation [11] among older people with dementia. Few studies have explored social participation in relation to technology use, and among those, there is a tendency to focus on novel technological innovations, such as gaming and self-monitoring, which have been limited by high attrition and low uptake in the everyday lives of older people with and without dementia [11,12]. Research based on how older people perceive social participation in a digitalized society found that digital technologies may be perceived as influencing social opportunities, access to services, and a sense of security; however, this research did not include people with dementia [13]. In order to develop technologies that are assistive for older people with and without dementia, it is important to gain further insights into how existing technologies that the older person already uses are assistive or inhibitive to their participation in the activities and places in public space, which they need and in which they wish to participate [14,15]. There are knowledge gaps about how the Everyday Technology (ET) that older people, with and without dementia, already use in their activities of daily living relates to their social participation.

**Figure 1:**
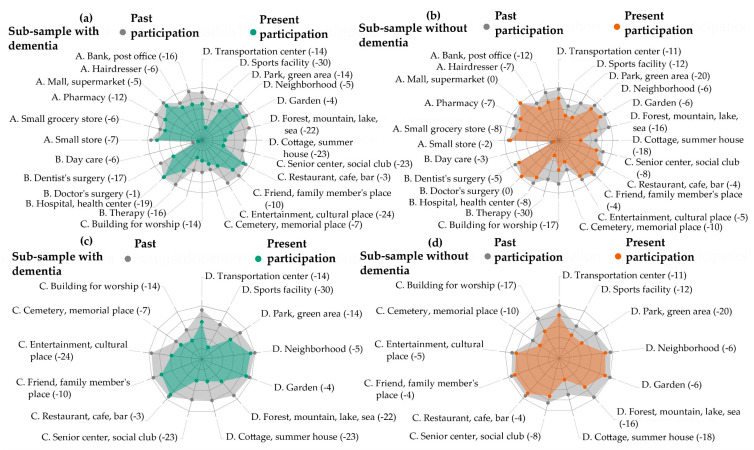
Visualizations to show changes in social participation between the past and present. Visualizations (**a**) and (**b**) show changes in past to present participation across all activities and places in the Participation in ACTivities and Places OUTside Home Questionnaire (ACT-OUT). Domains: (A) places for purchasing, administration, and self-care; (B) places for medical care; (C) places for social, spiritual, and cultural activities; (D) places for recreation and physical activity. Bracketed numbers correspond to decreases in participation. Green represents the sub-sample with dementia; orange represents the sub-sample without. Visualizations (**c**) and (**d**) show changes in past to present participation across ACT-OUT Domains C and D.

Social participation has been referred to as the person’s involvement in activities that provide interaction with others in society or the community’ [16]; however, there is no consensus definition of social participation, its component dimensions, or its relationship to technology [17]. Without a clear definition, understanding of ways to promote or to implement the benefits of social participation is inhibited [5,11]. For the purposes of this study, the data visualizations in Figure 1 were used to operationalize social participation as participation in places for social, spiritual, and cultural activities as well as those places for recreation and physical activities, in public space. Social participation in places in public space is therefore distinguishable from basic self-care or personal activities of daily living (e.g., getting dressed, bathing) which are predominantly performed within the home environment [16,18].

The data visualizations in Figure 1 draw on earlier research with the same United Kingdom (UK) sample, which did not explore differences between those people living with and without dementia [19]. The earlier study provides an impetus for this study’s more detailed investigation of the similarities and differences in social participation among older people with and without dementia. Here, the data visualizations show that older participants, particularly those with dementia, abandoned places and activities for social participation (e.g., entertainment, cultural places; senior center, social club; sports facility; cottage, summer house; forest, mountain, lake, sea) to a higher degree than other types of places, such as those for medical care (e.g., doctor’s surgery). Furthermore, participants with dementia showed a greater decrease between past and present social participation than participants without dementia. This corroborates earlier research among Swedish [20] and Swiss samples [21] and motivates the study’s focus on social participation. 

The study conceptualizes mainstream ETs as technologies that can be assistive but also inhibitive to social participation, among older people with and without dementia. ETs refer to the electronical, digital, and mechanical devices that typically exist in an older person’s environment and that are embedded in their habits and routines of everyday life [22]. Increasingly, these ETs include Information Communication Technologies (ICTs), such as the smartphone or a ticket machine, which are necessary to perform activities such as making an online transaction, accessing eHealth services, or getting a ticket for public transport [22]. Due to the embedded nature of technology in our everyday lives, it is believed that technology may be able to influence our performance of activities of daily living, including our social participation [8].

### Social Participation. Everyday Technology Use and Social Deprivation of the Living Environment: A Review of Key Issues

Studies indicate that dependency on ET use can be a reoccurring issue in activities inside and outside home, which people with cognitive impairments (e.g., due to mild-stage dementia, MCI, or stroke) just like all people in society, wish to master [23]. Earlier, research demonstrated that ET use can be inhibitive for people with dementia’s experience of accessibility in public space [24]. ET can be inhibitive to performing activities independently, for instance due to the replacement of human personnel in favor of self-service technologies [10,24]. Such technologies typically require a person to manage and remember several complex skills, including scanning a product, memorizing a code, and making a transaction using a credit card. Whilst earlier research highlights the ways that the dependency on ET use can diminish a person with dementia’s accessibility, more knowledge is required to understand how ET use specifically relates to not only accessibility but also social participation in public space. Building on the discourse, this study calls for the contextualization of social participation, in relation to ET use in places outside home.

A review of the literature indicates that inhibitors or barriers within one’s living environment should be considered when investigating social participation or technology use [18]. Research about participation in leisure activities demonstrated that an ability to freely choose to participate in activities and to negotiate barriers is a prerequisite for participation [25]. People with dementia have been shown to experience a greater range of barriers, which may inhibit participation and exacerbate the risk of social isolation if not fully considered [25]. Earlier research regarding potential barriers in one’s environment has found an association between digital engagement, specifically access and use of ICTs, and measures of social deprivation of the living environment based on the English Indices of Multiple Deprivation (IMD) [26]. Similarly, a study using the English Longitudinal Study of Ageing (ELSA) identified a relationship between loneliness and life satisfaction with relative deprivation of the living environment for older people [27]. There is an emerging evidence-base to suggest that social deprivation of the living environment is related to social participation, which requires further exploration.

The overall objective is to investigate social participation, in relation to total ET use outside home and social deprivation of the living environment, among participants with and without dementia in the UK sample. This motivates the following research questions:

- In what ways does social participation, as reported by older participants living with and without dementia, relate to total ET use outside home and social deprivation of the living environment?
- What are the motivators, considerations that require extra attention and, strategies for managing social participation of older people with and without dementia, in relation to the role of ET use outside home?

The first research question investigates potential associations between the reported social participation and total ET use outside home as well as social deprivation of the living environment using statistical methods. In order to address the second research question, a qualitative approach using content analysis of the free text responses is used to explore the motivators, considerations that require extra attention, and strategies for managing social participation, in relation to the role of ET use outside home. The structure of the mixed methods study is arranged according to an initial statistical analysis, followed by a content analysis of free text responses and, finally, an integration of the results in the discussion section. Data visualizations are incorporated throughout the study to illustrate key concepts and approaches used in the study.

## 2. Materials and Methods

### 2.1. Study Design and Ethics

A cross-sectional, convergent, mixed methods design was used. Quantitative data and free text responses were collected simultaneously. Different types of data were then integrated through analyses and interpretation of the findings [28]. The rationale for using a convergent mixed methods approach is that different types of data, e.g., numerical and free text responses, offer unique modes and levels of information, and collaboratively, can provide a more nuanced understanding of the research questions [28]. No formal power calculation was used due to the exploratory design of the study. Power calculations can be developed for future research based on the findings of this study. All participants gave their informed consent for inclusion before they participated in the study. The study was conducted in accordance with the Declaration of Helsinki, and ethics approval was granted from the Health Research Authority (IRAS project ID: 215654, REC reference: 17/SW/0091).

### 2.2. Participants

Data were collected across urban and rural environments in the UK, including five National Health Service (NHS) research sites (London, Cumbria, Greater Manchester regions). Participants with dementia (*n* = 64) were recruited through the NHS (e.g., memory clinics) and local community-based groups (e.g., memory cafes and local Alzheimer Associations). Participants without dementia (i.e., no known cognitive impairment) (*n* = 64) were recruited through local networks (e.g., community-based activity, faith or social groups). Participants with dementia had a diagnosis of mild-stage dementia, given by a physician based on the standardized Diagnostic and Statistical Manual of Mental Disorders, fourth edition, (DSM-IV) criteria, or as a major neurocognitive disorder, in the mild stage, according to the DSM-V [29,30]. The inclusion criteria specified that all participants were: (1) able to give informed consent to participate themselves, (2) aged 55 years or over, (3) living in ordinary housing in the community, (4) participating in activities outside home independently or with support, (5) using some ET, and (6) without vision or hearing impairments that could not be compensated via technical aids. Participants were given written and oral information about the study to consider on repeated occasions, before providing their written informed consent [31]. The rationale for including equally sized sub-samples of participants with and without dementia is based on research and policies that indicate a growing population of older people living and aging in their communities, which also includes a growing population of older people living with dementia [32].

### 2.3. Data Collection Procedure

Data collection was undertaken by two occupational therapists (first and sixth authors), through one-to-one interviews with participants in their homes or another location chosen by the participants, between May and December 2017. All participants had the option to be accompanied by a family member or a significant other as support but not for the purpose of proxy-reporting. Earlier research has revealed discrepancies in perspectives on social participation [18] and meaningful activities [33] among older people with cognitive impairments and caregivers. In order to be flexible to the needs of the participant and to ameliorate fatigue, interviews were divided into a maximum of three sessions over four weeks, lasting no longer than 90 min per session.

The interviews were comprised of four tools, used in the following order: (1) the Participation in ACTivities and Places OUTside the Home Questionnaire (ACT-OUT) [34], (2) the Montreal Cognitive Assessment (MoCA) [35], (3) a demographic questionnaire, (4) the Everyday Technology Use Questionnaire (ETUQ) [22].

(1) Part one of the ACT-OUT maps self-reported present participation in four domains: (A) places for purchasing, administration, and self-care, e.g., supermarket; (B) places for medical care, e.g., doctor’s surgery; (C) places for social, spiritual, and cultural activities, e.g., friend’s home; (D) places for recreation and physical activity, e.g., neighborhood. This study used the ACT-OUT to focus on domains C and D. A dichotomous yes or no answer was recorded for whether a person participated in the place or not. In part two of the ACT-OUT, the participant was asked to report in more detail about the activity they perform in one place, for each domain. This was based on a place where there had been no change in their participation. To capture detailed information about the activity performed in a place and the journey to and from the place, open-ended questions were used. Responses were written down verbatim by the interviewer and consisted of a couple of sentences. Examples of the questions include: “Picture yourself in a senior center or social club: what do you have to be careful about or pay extra attention to?” and “Imagine getting there: what do you have to be careful or pay extra attention to?”. Information about the development of the ACT-OUT and the functioning of its rating scale is described in earlier research [34].
(2) The MoCA [35] was performed with all participants in order to assess current levels of cognitive function to describe the sample.
(3) A non-standardized demographic questionnaire was used to gather information about participants, with respect to a range of demographic factors that may be relevant according to earlier research [36]. This included asking whether participants were living with a functional impairment (e.g., reduced fine motor skills or medical diagnoses such as diabetes). An Index of Multiple Deprivation (IMD) score was determined according to information about where the participants lived, which was checked according to LSOA listings on the Ministry of Housing, Community and Local Government website [37]. The IMD is a spatially disaggregated measure of relative deprivation applied to small geographic areas or neighborhoods in England, which are referred to as Lower-Super Output Areas (LSOAs) [37]. The IMD score comprises a weighted sum of seven sub-domains of deprivation: (1) income; (2) employment; (3) education, skills, and training; (4) health and disability; (5) crime; (6) access to housing and services; and (7) living environment. The IMD was used to contextualize the living environment of participants into ten equal groups (deciles), with one corresponding to the most deprived 10% of neighborhoods in England and ten representing the least deprived 10% of neighborhoods in England [37].
(4) The ETUQ was used to gather information about the total ET use outside home variable according to 49 ETs, including 16 ET which can be used outside home (e.g., ATM, self-service checkout) in addition to 33 portable ET that can be used both at home and outside home (e.g., smartphone, pedometer, eBook reader) [20]. The focus of this study is on the total ET use outside home; however, other studies provide descriptions about the use of each of the different types of ET [19]. In-depth information about the rating scale and validation of the ETUQ among different populations, including older people living with dementia, is available in earlier research [38,39].

### 2.4. Data Analysis

The convergent mixed methods approach involved three sequential steps: (1) statistical analysis of the ACT-OUT and ETUQ data; (2) content analysis of the free text responses from the ACT-OUT, through coding the data and collapsing the codes into categories and; (3) integration of the results from these two types of analyses in the discussion section, according to a graphical joint display [28]. Graphical joint displays are a type of visualization used to present mixed methods data in the form of a table or a figure. In this study, the graphical joint display was used to visualize insights beyond the information presented through separate results for the statistical or free text response data [40].

For the purposes of analysis, part one of the ACT-OUT was used to investigate social participation through two variables: (1) social participation in Domain C (social, spiritual and cultural activities; maximum six places) and (2) social participation in Domain D (recreation and physical activities; maximum seven places). To calculate total ET use outside home, for the two sub-samples, the scores for the 49 ETs were dichotomized based on them being used (1) or not used (0), and the dichotomous counts were summed.

**Figure 2:**
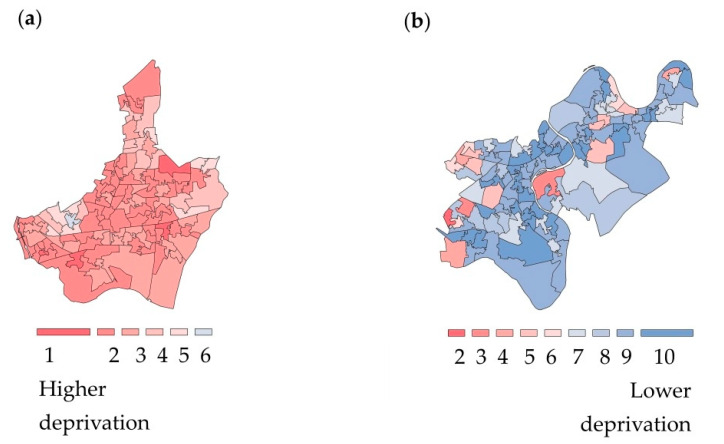
Visualizations of social deprivation of the living environment according to the Index of Multiple Deprivation. Choropleth maps of the Lower-layer super output areas by decile of the Index of Multiple Deprivation (IMD): (**a**) Barking and Dagenham, London; (**b**) Richmond upon Thames, London. According to the scale, one (red) refers to the most deprived 10% of neighborhoods, and ten (blue) represents the least deprived 10% of neighborhoods, in England. *Contains Ordinance OS data ^©^ Crown copyright and database right (2020)* [37].

Following an Exploratory Data Analysis approach, descriptive statistics and data visualizations were used to “isolate patterns and features of the data” [41]. Statistical analyses and the coding to create the data visualizations were performed in R (Version 3.6.1., R Foundation for Statistical Computing, Vienna, Austria) [42], with additional data exploration performed in Microsoft Power BI (Version 2.79.5768.1082, Microsoft Corporation, Washington, WA, USA) [43]. Microsoft Power BI is a business analytics and data visualization platform [43]. R is a programming language that was chosen due to its open access software environment for statistical computing and graphics [42]. For exploratory data analysis purposes, the IMD was used as a score for social deprivation of the living environment. The scores for social deprivation of the living environment were mapped to corresponding geospatial data about local authority districts from the neighborhoods where data was collected. R statistical software was then used to generate choropleth maps [44]. In the analysis, the color red was used to visualize the most deprived 10% of neighborhoods and blue to designate the least derived 10% of neighborhoods. Figure 2 presents examples of choropleths maps visualizing the variation in social deprivation of the living environment for two neighborhoods where data was collected. Data visualizations were created with color blind-friendly color palettes using ColorBrewer [45].

Differences between the two sub-samples in relation to demographic factors were tested. Normality testing indicated that the continuous variables (e.g., social participation in Domain C and Domain D, total ET use outside home, IMD) were not normally distributed, which motivated the use of non-parametric test statistics. Differences between the continuous variables were calculated (e.g., Mann–Whitney U-test) and correlations (Spearman’s rank correlation coefficients) were generated. Cohen’s guidelines for social sciences were used to interpret the strength of correlations: small = 0.1–0.3, medium = 0.3–0.5, and large effect = 0.5–1.0 [46]. For all analyses, the alpha level was set to 0.05.

Content analysis of the ACT-OUT free text responses was performed using the ATLAS.ti software program (Version 8, ATLAS.ti Scientific Software Development GmbH, Berlin, Germany) [47]. The rationale for performing a content analysis was to identify words, categories, and concepts within the free text responses and to make inferences about the contexts of their use [48]. No predefined coding themes were used, rather a coding system was developed based on the content of the data. Two researchers (first and third authors) coded a sub-sample of the data separately. The two researchers then engaged in critical discussions to review and refine the initial coding system and to reach a consensus draft code. The codes were collated and assigned to overall categories and sub-categories, with ongoing discussions until no new categories were identified.

## 3. Results

### 3.1. Statistical Results

#### 3.1.1. Participants

No statistically significant differences were identified in the demographic factors between the two sub-samples, with the exception of age, which was significantly higher among participants with dementia (*p* < 0.01); years of education, which were significantly lower for participants with dementia (*p* < 0.01); and the number of drivers, which was significantly lower among participants with dementia, compared to participants without dementia (*p* < 0.01) (Table 1). As expected, the MoCA score for participants with dementia was significantly lower than participants without dementia (*p* < 0.01).

#### 3.1.2. Social Participation in Relation to Total ET Use outside Home

Descriptive statistics showed that social participation in Domain C was significantly lower for participants with dementia (Median (*Md*) = 3.0) compared with participants without dementia (*Md* = 5.0; *U* = 1434.0; *Z* = −2.996; *p* = 0.003). However, the median social participation in Domain D was equal for the two sub-samples (*Md* = 5.0; *U* = 1900.0; *Z* = −0.719; *p* = 0.472) (Table 2). Total ET use outside home was significantly lower among participants with dementia (*Md* = 10.0) than participants without dementia (*Md* = 21.0; *U* = 556.50; *Z* = −7.114; *p* < 0.001).

#### 3.1.3. Social Participation in Relation to Social Deprivation of the Living Environment

Both sub-samples encompassed a minimum and maximum range across all ten deciles of the IMD. The IMD was lower but not significantly for participants with dementia (*Md* = 5.0) compared to participants without dementia (*Md* = 5.50; *U* = 2018.50; *Z* = −0.142; *p* = 0.887) (Table 1). This corresponds to a slightly higher proportion of participants with dementia living in more deprived areas of England, compared to participants without dementia, in this sample. Spearman’s rank correlations revealed no statistically significant association between social participation in Domain C and the total ET use outside home for participants with dementia (*R_s_* = 0.176, *p* = 0.164) or participants without dementia (*R_s_* = 0.181, *p* = 0.152). Conversely, a small but statistically significant association was found between social participation in Domain D and the total ET use outside home for participants with dementia (*R_s_* = 0.247, *p* = 0.049), and a small to medium, statistically significant association was identified for participants without dementia (*R_s_* = 0.343, *p* = 0.006). No statistically significant association was found between social participation in Domain C and IMD for participants with dementia (*R_s_* = 0.035, *p* = 0.785) or participants without dementia (*R_s_* = 0.157, *p* = 0.214). A small, statistically significant association was determined between social participation in Domain D and IMD for participants with dementia (*R_s_* = 0.267, *p* = 0.033) but not for participants without dementia (*R_s_* = 0.014, *p* = 0.911).

### 3.2. Content Analysis Results

The free text responses from participants with and without dementia correspond to their descriptions about motivators, considerations that require extra attention and strategies for managing social participation in Domain C and D.

#### 3.2.1. Purposeful Activity as a Motivator for Social Participation

Participants with and without dementia highlighted the importance of purposeful activities as a motivator for social participation. The degree of complexity attached to the purpose of social participation varied. Some places were associated with the purpose of performing a specific activity, for instance, walking in the park or eating in a restaurant. Other places were associated with repertoires of multiple activities such as going to a community center to meet people, see friends, pray, pass time, and for enjoyment (Table 2). In Domain C, the places and activities most frequently reported for participants with dementia (36.51%) were friend or family member’s place (e.g., visiting family to socialize and provide support), compared with entertainment or cultural places (e.g., watching a film at the cinema or visiting the library) for participants without dementia (23.43%). In Domain D, participants with dementia (28.33%) most frequently spoke about participation in their garden (e.g., take care of the garden, sit out and enjoy it) whilst participants without dementia (24.14%) commonly spoke about participation in the forest, mountain, lake, or seaside (e.g., go on a trip, walk, relax, alone, or with others).

#### 3.2.2. Doing the Journey and/or Activity with Other People as a Motivator for Social Participation

Participants with and without dementia described the journey as a natural continuation of the activity itself, rather than as two distinct parts. Participation in the journey and the activity were viewed as opportunities to socialize with other people including their spouse, family, friends, or as a group member. The rationale for doing the journey or activity together with other people differed between participants, e.g., due to a shared interest in the activity, to support each other with travel arrangements or for the pleasure of companionship as one participant with dementia explained, “only go with someone else—not so much fun otherwise”. Participation in the journey or activity with other people was viewed as a way of receiving or giving support to other people. A participant with dementia explained their motivation for participating with other people, “would not go without someone… would get lost”. Whilst the need for support with travel arrangements was a reoccurring issue for many participants, participants with dementia reported an increased dependency on the support of others for a range of reasons (e.g., following driving cessation).

#### 3.2.3. Contextual Factors that Require Extra Attention for Managing Social Participation

Contextual factors were spoken about in relation to considerations that require extra attention for social participation. Familiarity with people in one’s neighborhood was presented as an additional layer of support and security for social participation, among participants with and without dementia. A participant with dementia recalled that the support of one’s social context could be regarded as a buffer against problematic situations associated with ET (e.g., misplacing ET, forgetting to charge ET): “It’s good I have this place (café) and know all the people because if I ever got lost. One time my daughter couldn’t find me and I didn’t have my phone on me but she knew to check there”.

Conversely, participants also spoke about contextual factors in relation to perceived risks during social participation. Perceived risks ranged from the inconvenience of being distracted and not being able to escape conversations with people to concerns for one’s personal safety (e.g., intoxicated people, criminal behaviors, crowds). Participants reported that there would be no concerns at a place and that they felt able to socialize, if the behavior of other people was “considerate and reasonable”. 

Other considerations that required extra attention referred to the physical context, including ET. A participant without dementia explained that the alarms go off regularly which can be “disorientating and confusing”. Uneven walking surfaces and trip hazards also necessitated extra attention, and a participant without dementia described the challenge of participating in shared spaces outside home where other people cause clutter: “watch out for trip hazards—lots of baby gear cluttering up”. Problematic characteristics in the physical context were exacerbated by wet weather conditions, poor lighting, or temporal factors, leading to avoidance of going out in darkness at night-time. Urban and rural dwellers spoke about contextual factors to be aware of whilst travelling to and from places and activities outside home. Urban dwellers identified the need to pay extra attention to traffic, fumes, and parking, whilst several rural dwellers observed the need to consider other contextual factors, such as flooding, tides, or wildlife.

#### 3.2.4. Preparation and Wayfinding Involving ET Use for Managing Social Participation

Preparation and wayfinding were considered strategies to assist a person with managing whether they were able to engage in social participation or not. Management strategies frequently involved ET use at home as a preparatory activity, for subsequent participation outside home. For example, a participant without dementia described searching online for information about the accessibility of restaurants prior to making a reservation because they were responsible for organizing group meetings, “researches online to check [the restaurant] can accommodate (group’s) accessibility requirements prior to booking”.

Participants reported preparatory strategies for assisting them to find their way to a place and several of these strategies involved ET use (e.g., Global Positioning System (GPS)) for navigation and wayfinding. A participant with dementia explained that he had used his iPhone to find his way when he was lost, “use compass on my iPhone to navigate did get lost once but found my way”. However, a sense of anxiety was associated with the perceived need to plan and prepare. A participant without dementia explained that anxiety about the journey started with the act of planning and preparing: “Anxiety begins at the point of booking….” Similarly, a participant with dementia reported getting prepared long in advance of actually needing to leave the home to participate in places and activities outside home and the accompanying disagreements this caused with their spouse, “If I have an appointment or something that day I’m ready and prepared to go early in the morning even if it’s not till the afternoon, drives my wife mad—something we disagree on”.

Attitudes towards the need to plan and prepare for social participation, including the use of ET, conveyed nuanced meanings. Participants with and without dementia reported that they had encountered problems using ET for planning purposes or that they found ET limited in its ability to support them to manage social participation.

## 4. Discussion

The study identified perceived motivators, considerations that require extra attention, and management strategies underlying social participation, which supports earlier research indicating that older people express interest, motivation, and perceived wellbeing in relation to social participation [2]. A small, significant association was found between social participation and ET use outside home, in places for recreation and physical activities (Domain D) but not in places for social, spiritual, and cultural activities (Domain C). Social participation has been described as an abstract phenomenon that manifests in a variety of concrete social activities, such as visiting a place or using ET [51]. One explanation for the significant association found between social participation and ET use outside home, in places for recreation and physical activities (Domain D) but not in places for social, spiritual, and cultural activities (Domain C) may be that in places for social, spiritual, and cultural activities there is more of a social network to support the participant and thus, a lower dependency on ET use as a support for social participation. Participants with and without dementia tended to report being familiar or very familiar with places for social, spiritual, and cultural activities, for instance, having visited a specific place of worship or a community center for many years. Earlier research substantiates the importance of places, such as community centers or places of worship, for facilitating social connectedness and a sense of familiarity with one’s community [52]. A variety of factors may influence whether ET is used or not used in different places in public space. For instance, social and cultural norms may discourage technology use in cultural and spiritual places out of a sign of respect. More specifically, research about gender and age differences in mobile phone etiquette in social situations found that older people were more restrictive than younger people in their mobile phone use in places in public space, such as at church [53].

Alternatively, older participants may encounter a higher frequency of ET in the types of recreation and physical activities found in Domain D (e.g., ticket machines at transport centers or ET around a person’s neighborhood). It should be noted that whilst statistical associations demonstrate the existence of an association between ET use and social participation, detailed information about the direction and magnitude of the association is unknown. In fact, when the free text responses are investigated in relation to the statistical analyses, a degree of reciprocity emerges, whereby the participants describe not only how their ET use is assistive or inhibitive to their social participation but also how their motivators, considerations that require extra attention, and management strategies for social participation may be assistive or inhibitive to their ET use. Future research is needed to investigate this potential reciprocal relationship between social participation and ET use, as this may contribute knowledge to an emerging discourse about the reciprocal relationship between social participation and other factors such as health status, among older people [54].

The participants with and without dementia reported using mainstream ET, which they perceived as being familiar, in ways that assisted their participation in activities of daily living. Despite not directly asking about ET use, participants described ET use in relation to management strategies to assist them to plan and prepare before participating outside home (e.g., online information search) or for wayfinding purposes (e.g., GPS). This reinforces earlier research, which found that ET use is not only integral to the performance of the desired activity itself but also to preceding activities (e.g., managing public transport, using a ticket machine, operating an ATM). The preceding activities must be performed before the person can master their desired activity (e.g., socializing with friends, visiting the cinema) [23]. Research has shown that a person’s experience of public space or places outside home is linked to the type of preparatory activities that were required in the home environment. In earlier research, people with dementia reported that staying in control of participation in public space can be inhibited, and anxiety can be exacerbated because performing preparatory activities, such as finding and taking essential items including keys, a wallet, or a phone, drained their energy reserves even before departing the home environment to engage in social participation in places within public space [24]. The results suggest that greater support given to older people with and without dementia in preparatory and wayfinding stages, for example, through support using ET to help preserve energy reserves and mitigate anxiety, or the design and development of more usable wayfinding and preparatory applications within the mainstream ET that older people already use, may help to assist their ongoing social participation [15]. Research indicates that participatory approaches involving older people in the design of usable digital technologies may support social participation in their desired activities and enable them to live at home longer. However, further research among older people living with dementia is needed [13]. This substantiates research about how technology can help to offset the challenges that older people, particularly those with dementia, encounter. A scoping review about technology use to improve everyday occupations in older persons with mild dementia or mild cognitive impairment identified a number of different types of technology that may assist people with dementia to overcome barriers to their social participation [55]. These included time aids to compensate for difficulties in planning and managing one’s time, as well as tracking devices to support outdoor independence [55]. Whilst some positive effects were found for technology use among older people with dementia or mild cognitive impairment, there was a general lack of evidence about the ways in which technology use can help to offset barriers to social participation. 

A unique contribution of the study is its mixed methods approach which affirmed earlier research that older people with and without dementia perceive social participation in relation to their social and physical context [18] and that ET may enhance social participation [9]. However, the integration of mixed methods via visualization study also elucidated potential inconsistencies in statistical and content analyses from the ACT-OUT Questionnaire. The ACT-OUT Questionnaire asks about the place and the journey in two discrete parts; however, according to the free text responses, the journey was described as an extension of the activity and as an opportunity to socialize with other people for various reasons (e.g., companionship, to give or to receive support). The results confirm earlier research, which identified social support as a potential strategy for managing problematic situations in public space among people with dementia [24]. Knowledge about social factors, such as social support to travel, is particularly important for participants with dementia, to facilitate a balance between capacities and limitations [2] and to enable continued social participation, following changes in their mode of participation (e.g., following driving cessation). If as earlier research suggests, social participation is indeed a modifiable determinant of health and wellbeing among older people with and without dementia, the research suggests that it is equally important to consider strategies for promoting supportive social contexts for the journey as well as at the place and activity itself.

The results contribute to the definition of social participation by acknowledging social deprivation of the living environment. A small but statistically significant association was found between social deprivation of the living environment for participants with dementia, according to social participation in Domain D (places for recreation and physical activities). The neighborhood is an integral part of one’s living environment, and it was one of the places in Domain D that participants with and without dementia identified as a potential buffer to problematic situations in public space. Participants emphasized the importance of being familiar with one’s living environment, sometimes through the lens of their neighborhood. A recent systematic meta-synthesis on the experience of lived space in people with dementia found that familiarity was necessary in order to facilitate a sense of being at home for people with dementia, as well as supporting them to maintain their social relationships, identity, autonomy, security, and privacy [56].

The association between social participation and the social deprivation of the living environment builds on earlier research indicating that social deprivation of the living environment is linked to loneliness and life satisfaction among older people [28]. Earlier research underlines the importance of being aware of a potential relationship between the contextual aspects of the living environment and technology use for enabling older people to live and age in their communities [57]. Peek et al.’s conceptual model of factors influencing the level of technology use by older people who are aging-in-place conceptualizes technology as embedded in the personal, social, and physical context of the older person [57]. Their conceptual model underlines a need to be aware of the contextual and psychological factors in the older person’s environment in order to facilitate their aging-in-place through technology use [57]. This study builds on such earlier research concerning older people in general, by exploring contextual factors such as the social deprivation of the living environment in relation to technology use, among older people with and without dementia.

Generalizability of the statistical results is limited by the sample size and necessitates future research using a larger sample size [27]. Uniquely, this study highlighted the potential susceptibility of people with dementia to the effects of social deprivation of the living environment on social participation; however, this study does not infer any causal relationship. It should be noted that the free text responses, including descriptions of the social environment or the proportions of people participating in specific types of activities, are based on a convenience sample. The convenience sample raises potential issues of confounding for affluence and geographical location (e.g., urban compared with rural deprivation), and therefore, a sampling frame which is cognizant of such issues, for instance considering social deprivation of the environment from the start, is recommended for subsequent research.

## 5. Conclusions

This study utilized a mixed methods approach in order to investigate not only the statistical relationship between social participation, Everyday Technology use, and social deprivation of the living environment but also to explore the underlying perceived motivators, considerations requiring extra attention, and strategies for managing social participation which may involve ET use for this population. The results indicate the importance of ET use, particularly for preparation and wayfinding strategies, as a means of promoting and maintaining social participation, among older people with and without dementia. Insights into perceived motivators, considerations to pay extra attention to, and management strategies for social participation should be acknowledged, in order to realize the health benefits of social participation among older people with and without dementia. Moreover, the results indicate ways that ET use is perceived as being assistive to social participation but also the need to contextualize ET use and social participation according to other barriers such as social deprivation of the living environment, especially among people with dementia. Such knowledge can contribute to digital accessibility through guiding the design and development of more accessible mainstream, ET, and technological environments. Future longitudinal research exploring the relationship between ET use and the way that older people with dementia participate in places in public space may provide additional insights into the mechanisms underlying ET use and social participation, as well as other forms of participation, over time.

## Figures and Tables

**Table 1:** Demographic characteristics, Montreal Cognitive Assessment (MoCA) and Index of Multiple Deprivation Score (IMD).

Characteristics	Sub-Sample with Dementia (*n* = 64)	Sub-Sample without Dementia (*n* = 64)
Age, Years **		
Median (Min, Max) IQR ^1^	79.0 (62.0, 96.0) 74.0–83.0	71.0 (55.0, 89.0) 64.0–80.8
Gender, *n* (%)		
Female	29 (45.3)	34 (53.1)
Male	35 (54.7)	30 (46.9)
Living Arrangement, *n* (%)		
Cohabiting	39 (60.9)	40 (62.5)
Lives Alone	25 (39.1)	24 (37.5)
Geography, *n* (%)		
Urban	51 (79.7)	47 (73.4)
Rural	13 (20.3)	17 (26.6)
Education, Years **		
Median (Min, Max) IQR	11.0 (7.0, 21.0) 10.3–13.0	13.0 (9.0, 19.0) 11.0–16.0
Ethnicity, *n* (%)		
White	56 (87.5)	49 (76.5)
Mixed/Multiple Ethnic Group	0 (0.0)	2 (3.1)
Asian/Asian British	1 (1.6)	9 (14.1)
Black/African/Caribbean/ Black British	5 (7.8)	3 (4.7)
Other Ethnic Group	2 (3.1)	1 (1.6)
Driving, *n* (%) **		
Driving	26 (40.6)	46 (71.9)
Not Driving	38 (59.4)	18 (28.1)
Functional Impairment, *n* (%)		
Functional Impairment	54 (84.4)	56 (87.5)
No Functional Impairment	10 (15.6)	8 (12.5)
MoCA Score **		
Median (Min, Max) IQR	21.0 (12.0, 28.0) 18.0–23.0	26.0 (21.0, 30.0) 25.0–28.5
Index of Multiple Deprivation (IMD)		
Median (Min, Max) IQR	5.0 (1.0, 10.0) 3.5–8.0	5.5 (1.0, 10.0) 4.0–7.0

^1^ IQR: Interquartile range. ** Significant group differences (*p* < 0.01). One to ten deciles of the Index of Multiple Deprivation (IMD); one refers to the most deprived 10% of neighborhoods in England. Rural-Urban classification according to the Office of National Statistics (ONS) (2013) [49]. Ethnicity classification from the ONS Census (2011) [50].

**Table 2:** Graphical joint display to visualize the integration of mixed methods results.

Domain	Variables		Participants with Dementia (*n* = 64)	Participants without Dementia (*n* = 64)
Domain C (Social, Spiritual and Cultural Places)	Total social participation (places = 6)		Median: 3.0; Min–Max: 1.0–6.0; IQR: 3.0–5.0	Median: 5.0; Min–Max: 1.0–6.0; IQR: 3.0–5.0
	Places in Domain C and examples of activities performed in the places	**Places:** 1. Friend or family member’s place 2. Restaurant, café, bar 3. Senior center, social club 4. Building for worship 5. Cemetery, memorial place 6. Entertainment, cultural place	**Activities:** 1. Drive to daughter’s place, a trip 2. Going into the village, sit down, cup of tea, chat 3. Dementia care group and socialize, art group, crossword, singalong 4. Socialize, worship, church duties 5. N/A 6. Go and watch live music	**Activities:** 1. Socialize and see grandchildren 2. Breakfast different place each week 3. Gujarati center. 150 people go. Meet people there, see friends, prayer, pass time, enjoys herself 4. Worship, play guitar, pray, enjoy time with dear friends 5. Walk and look around cemetery 6. See a concert with a friend
	Association between Social participation and: i. Total Everyday Technology use outside home; ii. IMD		i. No significant association (*R_s_* = 0.176; *p* = 0.164) ii. No significant association (*R_s_* = 0.035, *p* = 0.785)	i. No significant association (*R_s_* = 0.181; *p* = 0.152) ii. No significant association (*R_s_* = 0.157, *p* = 0.214)
Domain D (Places for Recreation and Physical Activities)	Total social participation (places = 7)		Median: 5.0; Min–Max: 0.0–7.0; IQR: 3.0–6.0	Median: 5.0; Min–Max: 1.0–7.0; IQR: 4.0–6.0
	Places in Domain D and examples of activities performed in the places	**Places:** 1. Garden 2. Park, green area 3. Forest, mountain, lake, sea 4. Cottage, summer house 5. Neighborhood 6. Sports facility 7. Transportation center	**Activities:** 1. Garden, admire the garden and the birds 2. Goes with granddaughter so she can play on the swings 3. Walk the dog along the seafront for a friend 4. Visit son and grandchildren and have a break 5. Neighborhood, sometimes walk and get fresh air 6. Seated exercises 7. Traveling	**Activities:** 1. Sit there with wife and cat 2. Go to park, people watch, go to café, sit on bench, walk 3. Walk and enjoy the scenery and navigate 4. Has 3 days at the timeshare cottage 5. Walking around, thinking alone. Enjoy exercise, time to think, fresh air 6. Swimming, 70 lengths 7. Get underground or buses
	Association between Social participation and: i. Total Everyday Technology use outside home; ii. IMD		i. Small significant association (*R_s_* = 0.247; *p* = 0.049) ii. Small significant association (*R_s_* = 0.267, *p* = 0.033)	i. Small-medium significant association (*R_s_* = 0.343; *p* =0.006) ii. No significant association (*R_s_* = 0.014, *p* = 0.911)
Domain C and D	Motivators for social participation		Purposeful activity; doing the journey and/or activity with other people	
	Considerations that require extra attention for social participation		Contextual factors	
	Strategies for managing social participation		Preparation and wayfinding	
